# Supplementary material for: Recombination and mutational robustness in neutral fitness landscapes
Source: PLoS Comput Biol. 2019 Aug 15;15(8):e1006884. doi: 10.1371/journal.pcbi.1006884 (PMC6711544; doi:10.1371/journal.pcbi.1006884)
Supplement: S1 Appendix — (PDF) [file pcbi.1006884.s001.pdf]

Recombination and mutational robustness in neutral fitness landscapes: Supplementary appendix

Alexander Klug,[1] Su-Chan Park,[2] and Joachim Krug[1]

[1]Institute for Biological Physics, University of Cologne, Cologne, Germany

[2]Department of Physics, The Catholic University of Korea, Bucheon, Republic of Korea

I. VISUALIZATION OF FITNESS LANDSCAPES AS NETWORKS

In order to visualize random neutral fitness landscapes with more than two loci we make use of a network representation, where genotypes that differ by a single mutation are connected by an edge. Nodes of the network then represent genotypes, which are arranged according to a spring layout that is based on a Fruchterman-Reingold force-directed algorithm [1]. To describe this algorithm briefly, nodes are made to repel each other, which is counteracted by edges that function as springs. This leads to a process of spring-force relaxation that arrives at an equilibrium state which in turn is used for the node positions. The equilibrium state is characterized by clustering of highly connected regions of nodes. Therefore this algorithm is only useful if not all nodes have the same number of edges. Hence edges attached to lethal genotypes are deleted. This leads to a network in which only viable genotypes that differ by a single mutation are connected. Lethal genotypes are off the grid and create a ring of repelled nodes.

II. TWO-LOCUS MODEL WITH UNIDIRECTIONAL MUTATION

Following Nowak *et al.* [2], we consider the two-locus model with unidirectional mutations from allele 0 to allele 1 at rate μ and one-point crossover at rate r. Based on the relation

$$q_0 = \frac{r}{4\tilde{\mu}} q_1^2, \quad \tilde{\mu} = \frac{\mu}{1-\mu} \tag{A1}$$

between the lumped genotype frequencies after selection, the expression

$$M = q_0 + \frac{1}{2} q_1 = 1 - \frac{\tilde{\mu}}{r}\left(\sqrt{1+\frac{r}{\tilde{\mu}}} - 1\right) \tag{A2}$$

can be derived for the mutational robustness after selection. For $r \to 0$ this reduces to $M = \frac{1}{2}$ independent of μ, which is smaller than the value $M = \frac{2}{3}$ expected for a random distribution over the viable genotypes ($q_0 = \frac{1}{3}, q_1 = \frac{2}{3}$). In the absence of recombination, the unidirectional mutations drive the entire population into the least robust genotypes (0,1) and (1,0), such that $q_0 = 0$ and $q_1 = 1$. On the other hand, for $r = 1$ Eq (A2) becomes $M = (1 + \sqrt{\mu})^{-1}$, which can be compared to the corresponding expression

$$M = \frac{m}{1 - f_2} = \frac{2}{2 - \mu + \sqrt{\mu^2 + 4\mu}} \tag{A3}$$

obtained from Eq (17) of the main text. The two expressions coincide for $\mu \to 0$, but for larger μ the bidirectional model has higher robustness, because both selection and recombination contribute to focusing the population onto the robust genotype (0,0) (Fig A1).

III. MUTATIONAL ROBUSTNESS ON THE MESA LANDSCAPE WITH COMMUNAL RECOMBINATION

In this section, we calculate the mutational robustness in equilibrium for the mesa landscape, using the communal recombination scheme [3]. Since fitness depends only on the Hamming distance from the wild type, the equilibrium allele-frequency distribution at each locus is the same after mutation. In the following we denote the (equilibrium) frequency of allele 0 (1) after the mutation step by π_0 ($\pi_1 = 1 - \pi_0$). Then the equilibrium frequency f_σ^* of a genotype σ after recombination becomes

$$f_\sigma^* = \pi_0^{L-n} \pi_1^n, \tag{A4}$$

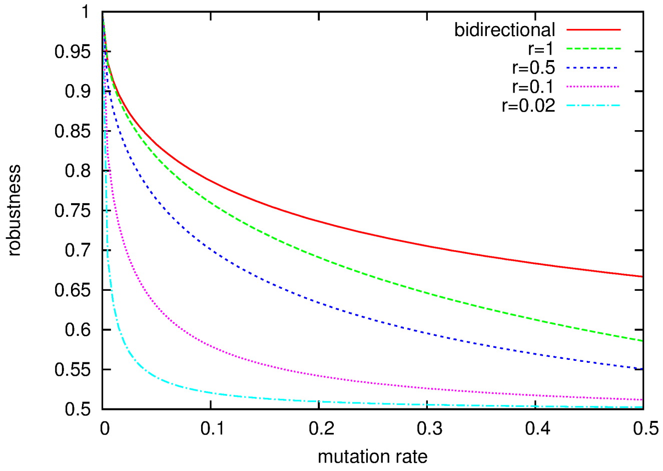

Fig A1. **Mutational robustness in the two-locus model with unidirectional mutations**. The figure shows the mutational robustness after selection obtained for the unidirectional mutation scheme, Eq (A2), as function of μ for different r. For comparison the corresponding result Eq (A3) for the bidirectional mutation scheme with $r = 1$ is also depicted.

where n is the Hamming distance from the wild type. The lumped frequency of all genotypes in the class with n mutations is then given by

$$f_n = \binom{L}{n} \pi_0^{L-n} \pi_1^n. \tag{A5}$$

Denoting the corresponding lumped frequency after selection by q_n and using the mesa landscape defined in Eq (28) of the main text, we get

$$q_n = \begin{cases} f_n \bar{w}^{-1}, & n \leq k, \\ 0, & n > k, \end{cases} \tag{A6}$$

where $\bar{w} = \sum_{n=0}^{k} f_n$ is the mean fitness. The lumped frequency p_n after mutation then satisfies

$$p_d = \sum_{n=0}^{L} \mu(d|n) q_n, \tag{A7}$$

where $\mu(d|n)g_d$ is the probability that a mutation changes the Hamming distance from n to d. The p_d in turn determine the allele frequency after mutation through

$$\pi_1 = \frac{1}{L} \sum_{d=0}^{L} d p_d = \frac{1}{L} \sum_d d \sum_n \mu(d|n) q_n = \frac{1}{L} \sum_n h(n) q_n, \tag{A8}$$

where $h(n) = \sum_d \mu(d|n) d$ is the average Hamming distance of a mutant generated from a genotype with Hamming distance n. One can easily calculate $h(n)$ for the mutation scheme Eq (4) of the main text, which yields

$$h(n) = n(1-\mu) + (L-n)\mu = L\mu + (1-2\mu)n. \tag{A9}$$

This expression has a simple interpretation: On average a fraction $1-\mu$ of the n mutated sites is not mutated, and a fraction μ of the $L-n$ non-mutated sites aquires a new mutation. Inserting Eq (A9) into Eq (A8), we finally obtain

$$\begin{aligned} \pi_1 &= \frac{1}{L} \sum_{n=0}^{k} \left[L\mu + (1-2\mu)n\right] q_n = \mu + \frac{1}{L}(1-2\mu) \sum_{n=0}^{k} n q_n = \mu + \frac{1}{L\bar{w}}(1-2\mu)\left(L\pi_1 - \sum_{n=k+1}^{L} n f_n\right) \\ &= \mu + \frac{\pi_1}{\bar{w}}(1-2\mu) - \frac{1}{L\bar{w}}(1-2\mu) \sum_{n=k+1}^{L} n f_n, \end{aligned} \tag{A10}$$

where we have used that

$$\sum_{n=0}^{L} n f_n = \sum_{n=0}^{L} n p_n = L\pi_1, \tag{A11}$$

because the allele frequency is not changed by recombination.

Up to now, everything is exact. It is a formidable, if not impossible, task to find an exact solution of Eq (A10), so we will solve the problem approximately for small μ. Since μ is small, it is plausible to assume that $\pi_1 \ll 1$ as well. Under this assumption, we can find an approximate expression for $\bar{w}$ as follows:

$$\begin{aligned}\bar{w} = \sum_{n=0}^{k} f_n = 1 - \sum_{n=k+1}^{L} f_n &\approx 1 - \binom{L}{k+1}\pi_1^{k+1}(1-\pi_1)^{L-k-1} - \binom{L}{k+2}\pi_1^{k+2}(1-\pi_1)^{L-k-2}\\ &\approx 1 - \binom{L}{k+1}\pi_1^{k+1} + (L-k-1)\binom{L}{k+1}\pi_1^{k+2} - \binom{L}{k+2}\pi_1^{k+2}\\ &= 1 - \binom{L}{k+1}\pi_1^{k+1} + (k+1)\binom{L}{k+2}\pi_1^{k+2} \equiv 1 - C_1\pi_1^{k+1} + (k+1)C_2\pi_1^{k+2},\end{aligned} \tag{A12}$$

where we have kept terms up to order π_1^{k+2}, $C_i = \binom{L}{k+i}$ $(i = 1, 2)$, and $1/j!$ should be interpreted as 0 if j is a negative integer. Note that the above formula is actually exact for $k \geq L - 2$.

Now we approximate Eq (A10) term by term. First, we get

$$\frac{\pi_1}{\bar{w}}(1-2\mu) \approx \pi_1\left[1 + C_1\pi_1^{k+1} - (k+1)C_2\pi_1^{k+2}\right](1-2\mu) \approx \pi_1 - 2\mu\pi_1 + C_1\pi_1^{k+2}, \tag{A13}$$

where we have kept terms up to π_1^{k+2} and $\mu\pi_1$. Second, we get

$$\begin{aligned}\frac{1-2\mu}{\bar{w}}\sum_{n=k+1}^{L} n f_n &\approx \left[1 + C_1\pi_1^{k+1} - (k+1)C_2\pi_1^{k+2}\right](1-2\mu)\left[(k+1)C_1\pi_1^{k+1}(1-(L-k-1)\pi_1) + (k+2)C_2\pi_1^{k+2}\right]\\ &\approx (k+1)C_1\pi_1^{k+1} - k\frac{L!}{(k+1)!(L-k-2)!}\pi_1^{k+2}.\end{aligned} \tag{A14}$$

Accordingly, we arrive at

$$\begin{aligned}\pi_1 &\approx \mu + \pi_1 - 2\mu\pi_1 + C_1\pi_1^{k+2} - \frac{k+1}{L}C_1\pi_1^{k+1} + k\frac{(L-1)!}{(k+1)!(L-k-2)!}\pi_1^{k+2}\\ &= \pi_1 + \mu - 2\mu\pi_1 - \binom{L-1}{k}\pi_1^{k+1} + (L-k)\binom{L-1}{k}\pi_1^{k+2},\end{aligned} \tag{A15}$$

that is,

$$\mu \approx B^{-(k+1)}\pi_1^{k+1} + 2\mu\pi_1 - (L-k)B^{-(k+1)}\pi_1^{k+2}, \tag{A16}$$

where $B = [k!(L-k-1)!/(L-1)!]^{1/(k+1)}$. Since the leading behavior of π_1 is $B\mu^{1/(k+1)}$, we set

$$\pi_1 = B\mu^{1/(k+1)}(1+g), \tag{A17}$$

where $g = o(1)$. Inserting Eq (A17) into Eq (A16) and expanding up to the leading order in g, we obtain

$$\begin{aligned}\mu &\approx \mu(1+g)^{k+1} + 2B\mu^{(k+2)/(k+1)} - (L-k)B\mu^{(k+2)/(k+1)}\\ &\approx \mu + \mu(k+1)g + (2+k-L)B\mu\mu^{1/(k+1)},\end{aligned} \tag{A18}$$

which yields

$$g \approx \frac{L-k-2}{k+1}B\mu^{1/(k+1)}. \tag{A19}$$

Therefore the mutational robustness becomes

$$\begin{aligned}
m &= \sum_{n=0}^{k-1} f_n + \frac{k}{L} f_k = 1 - \sum_{n=k+2}^{L} f_n - \frac{L-k}{L} f_k - f_{k+1} \approx 1 - \frac{L-k}{L}\binom{L}{k}\left[\pi_1^k - (L-k)\pi_1^{k+1}\right] - \binom{L}{k+1}\pi_1^{k+1} \\
&= 1 - \frac{(L-1)!}{k!(L-k-1)!}\pi_1^k + \frac{(L-1)!}{(k+1)!(L-k-1)!}(kL - k^2 - k)\pi_1^{k+1} \\
&= 1 - B^{-(k+1)}\pi_1^k + B^{-(k+1)}\pi_1^{k+1}\frac{kL-k^2-k}{k+1} \approx 1 + \mu\frac{kL-k^2-k}{k+1} - B^{-(k+1)}\pi_1^{k+1}\pi_1^{-1} \\
&\approx 1 + \mu\frac{kL-k^2-k}{k+1} - \mu[1+(k+1)g](1-g)\mu^{-1/(k+1)}B^{-1} \approx 1 + \mu\frac{kL-k^2-k}{k+1} - \mu^{k/(1+k)}(1+kg)B^{-1} \\
&= 1 + \mu\frac{kL-k^2-k}{k+1} - \mu^{k/(1+k)}B^{-1} - \mu^{k/(1+k)}kgB^{-1} \approx 1 - \mu^{k/(1+k)}B^{-1} + \mu\frac{kL-k^2-k}{k+1} - \mu\frac{k(L-k-2)}{k+1} \\
&= 1 - \binom{L-1}{k}^{1/(k+1)}\mu^{k/(k+1)} + \mu\frac{k}{k+1}. \qquad \text{(A20)}
\end{aligned}$$

If $L \gg k$, m can be approximated as

$$m \approx 1 - (L\mu)^{k/(k+1)}(k!)^{-1/(k+1)} + \mu\frac{k}{k+1}. \qquad \text{(A21)}$$

IV. MUTATIONAL ROBUSTNESS ON THE MESA LANDSCAPE IN THE ABSENCE OF RECOMBINATION

Here we calculate the mutational robustness for the mesa landscape in the absence of recombination and under the assumption that the mutation rate is small. Here this is taken to imply that the genome-wide mutation rate $U \equiv L\mu \ll 1$, which implies that multiple mutations are negligible in the mutation step. Using the same notation as before, the lumped equilibrium frequencies after mutation f_n and after selection q_n then satisfy the relations

$$\bar{w} = \sum_{n=0}^{k} f_n, \quad q_n = \frac{f_n}{\bar{w}}, \quad f_n = (1-U)q_n + U\frac{L-n+1}{L}q_{n-1} + U\frac{n+1}{L}q_{n+1}, \qquad \text{(A22)}$$

where $q_n = 0$ for $n > k$ and $q_{-1} = 0$. Since $f_n = 0$ for $n > k+1$, we have

$$\bar{w} = 1 - f_{k+1} = 1 - U\frac{L-k}{L}q_k. \qquad \text{(A23)}$$

This yields a closed set of equations for the q_n, which reads

$$q_n\left[1 - U\left(1 - \frac{k}{L}\right)q_k\right] = (1-U)q_n + U\frac{L-n+1}{L}q_{n-1} + U\frac{n+1}{L}q_{n+1} \qquad \text{(A24)}$$

or

$$\frac{n+1}{L}q_{n+1} = M_k q_n - \frac{L-n+1}{L}q_{n-1}, \qquad \text{(A25)}$$

with

$$M_k = 1 - \frac{L-k}{L}q_k = \sum_{n=0}^{k-1} q_n + \frac{k}{L}q_k = 1 - \left(1 - \frac{k}{L}\right)q_k. \qquad \text{(A26)}$$

Note that M_k can be interpreted as mutational robustness measured before mutation and after selection. Interestingly, q_n's do not depend on U if no multiple mutations are allowed. Since mutational robustness after mutation is given by

$$\begin{aligned}
m &= \sum_{n=0}^{k-1} f_n + \frac{k}{L}f_k = 1 - f_{k+1} - \frac{L-k}{L}f_k = \bar{w}\left(1 - \frac{L-k}{K}q_k\right) = M_k\bar{w} \\
&= M_k - UM_k(1-M_k) = M_k(1-U) + UM_k^2, \qquad \text{(A27)}
\end{aligned}$$

it is sufficient to find M_k.

Defining $\xi_n \equiv (2L)^{n/2}\binom{L}{n}^{-1} q_n/q_0$ and $y \equiv M_k\sqrt{L/2}$, we obtain from (A25)

$$\left(1-\frac{n}{L}\right)\xi_{n+1} = 2y\xi_n - 2n\xi_{n-1}. \tag{A28}$$

We write down the first few terms for later purposes,

$$\xi_0 = 1, \quad \xi_1 = 2y, \quad \xi_2 = (4y^2-2)\frac{L}{L-1}. \tag{A29}$$

If $n/L \ll 1$, Eq (A28) is approximated as

$$\xi_{n+1} = 2y\xi_n - 2n\xi_{n-1}, \tag{A30}$$

which is the recursion relation of the Hermite polynomials $H_n(y)$. Since $\xi_0 = H_0$ and $\xi_1 = H_1$ for any L, we find the approximate solution for ξ_n as $\xi_n = H_n(y)$ for $n \ll L$. If $k/L \ll 1$, the Hermite polynomial becomes an accurate solution for all n. Since $\xi_{k+1} = 0$ by definition and $\xi_n > 0$ for $n \le k$, y should be the largest solution of the equation

$$H_{k+1}(y) = 0. \tag{A31}$$

If we denote the largest zero of Eq (A31) by $\sqrt{y_k/2}$, we thus conclude

$$M_k = \sqrt{\frac{y_k}{L}} + o(L^{-1/2}). \tag{A32}$$

The first few zeros are given by

$$y_1 = 1, \quad y_2 = 3, \quad y_3 = 3+\sqrt{6}, \quad y_4 = 5+\sqrt{10}. \tag{A33}$$

The approximation can be compared to the exact solutions for M_k which have been obtained up to $k=4$ by solving Eq (A22),

$$\begin{aligned}
M_1 &= \frac{1}{\sqrt{L}}, \quad M_2 = \frac{\sqrt{3L-2}}{L} = \sqrt{\frac{3}{L}} + O(L^{-3/2}),\\
M_3 &= \frac{\sqrt{3L-4+\sqrt{6L^2-3L+16}}}{L} = \left(\frac{3+\sqrt{6}}{L}\right)^{1/2} + O(L^{-3/2}),\\
M_4 &= \frac{\sqrt{5L-10+\sqrt{10L^2-5L+76}}}{L} = \left(\frac{5+\sqrt{10}}{L}\right)^{1/2} + O(L^{-3/2}),
\end{aligned}$$

which are indeed consistent with Eq (A32) and the first four y_k's in Eq (A33). Using Eq (A27) the robustness after mutation is then given by

$$m \approx \sqrt{\frac{y_k}{L}}(1-U) + U\frac{y_k}{L}. \tag{A34}$$

Now we consider the case of large k. If we still assume $1 \ll k \ll L$, the above approximation is valid. Since the asymptotic behavior of the largest zero of $H_n(x)$ is $\sim \sqrt{2n+1}$ [4, p. 132], we find $y_k \sim 4k$, which gives

$$m \approx 2\sqrt{\frac{k}{L}}(1-U). \tag{A35}$$

The approximation leading to Eq (A32) is however not valid if k/L remains finite as $L \to \infty$. To treat this problem, we may refer to previous work on the mesa landscape [5] that makes use of a maximum principle for permutation-invariant fitness landscapes [6]. This principle states that the stationary population mean fitness $\bar{w}$ is given by

$$\bar{w} = \max_{x\in[0,1]}\left\{\omega(x) - U\left[1-2\sqrt{x(1-x)}\right]\right\}, \tag{A36}$$

where $\omega(x) = \lim_{L\to\infty} w_{xL}$ is the limiting value of the fitness of a genotype with $n = xL$ mutations. To account for the fact that genotypes with more than k mutation are lethal, the fitness function has to be taken to be $\omega(x) = 1$ if $x \leq x_0 \equiv k/L$ and $\omega(x) = -\infty$ if $x > x_0$, which is slighlty different from the setting of Ref. [5]. Nevertheless the result for the stationary fitness is the same,

$$\bar{w} = \begin{cases} 1 - U\left[1 - 2\sqrt{x_0(1-x_0)}\right], & \text{if } x_0 < 1/2, \\ 1, & \text{if } x_0 \geq 1/2. \end{cases} \tag{A37}$$

Combining Eqs (A23) and (A26) we see that $M_k = 1 - U^{-1}(1-\bar{w})$, and therefore

$$M_k = \begin{cases} 2\sqrt{x_0(1-x_0)}, & \text{if } x_0 < 1/2, \\ 1, & \text{if } x_0 \geq 1/2. \end{cases} \tag{A38}$$

Note that the leading behavior of M_k for small x_0 is the same as the Hermite polynomial solution Eq (A35).

V. RECOMBINATION WEIGHT ON THE MESA LANDSCAPE WITH UNIFORM CROSSOVER

In order to efficiently compute the recombination weight for uniform crossover on the mesa landscape, one has to exploit the permutation invariance of the landscape. In the following we denote the recombination weight λ_σ of genotype σ as $\lambda(L, a, k, r)$, since it is fully defined by the sequence length L, the mesa width k, the Hamming distance $a \equiv d_\sigma$ to the wild type and the recombination rate r. To start with we first note that the Hamming distances between an offspring genotype σ and its parent genotypes κ, τ also determine the Hamming distance between both parent genotypes through the relation [7]

$$d(\sigma,\kappa) + d(\sigma,\tau) = d(\kappa,\tau). \tag{A39}$$

For the following it is convenient to introduce the variables i and j which represent the Hamming distance $d(\sigma,\kappa)$ and $d(\sigma,\tau)$, respectively. Eq (A39) is useful since the Hamming distance $i+j$ between the parent genotypes determines their number of possible distinct offspring genotypes through recombination. Hence the probability that the offspring genotype σ is generated by two genotypes at distance i and j is given by

$$\frac{1}{2^{i+j}} r + \frac{1-r}{2}\left(\delta_{i0} + \delta_{j0}\right), \tag{A40}$$

where the second term includes the possibility of no recombination for which at least one of the parent genotypes needs to be the same as the offspring genotype, see also Eq (6) of the main text. Next we consider the number of genotypes at Hamming distance i and j as well as their respective fitness. The number of potential parent genotypes at Hamming distance i is given by $\binom{L}{i}$ which can be rewritten as

$$\binom{L}{i} = \sum_{x=0}^{i} \binom{a}{x}\binom{L-a}{i-x} = \sum_{x=\max(0,i+a-L)}^{\min(i,a)} \binom{a}{x}\binom{L-a}{i-x}. \tag{A41}$$

We make use of the fact that in order to create a genotype at distance i, we can mutate x out of a 1-alleles and $i-x$ out of $L-a$ 0-alleles from the offspring genotype for which the number of arrangements is given by a binomial coefficient. Since the sum might contain zero terms we can restrict the summation range further. Through this expression it is possible to relate to each genotype its fitness which is given by

$$w(k, (a-x) + (i-x)) = \theta(k - (a-x) - (i-x)), \tag{A42}$$

where $(a-x) + (i-x)$ denotes the number of 1-alleles in the parent genotype and θ is the Heaviside step function with $\theta(0) = 1$. After choosing a parent genotype at distance i the remaining number of suitable parent genotypes at Hamming distance j is thus given by

$$\sum_{y=0}^{j} \binom{a-x}{y}\binom{L-a-(i-x)}{j-y} = \sum_{y=\max(0,j+a-L+i-x)}^{\min(j,a-x)} \binom{a-x}{y}\binom{L-a-(i-x)}{j-y}, \tag{A43}$$

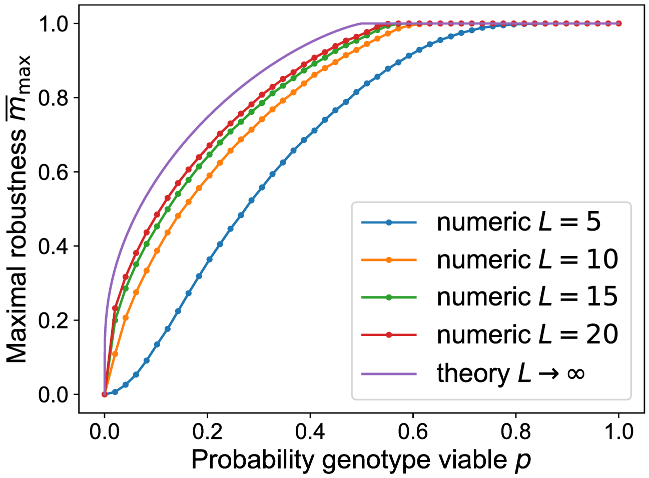

Fig A2. **Maximal degree of the viable network in the percolation landscape.** The figure shows numerical results for the expected maximal degree of a viable genotype in percolation landscapes of different size L. For $L \to \infty$ the results converge to the solution z^* of the equation $s_p(z^*) = \ln 2$, where $s_p(z)$ is given in Eq (A47).

with fitness

$$w(k, (a-y) + (j-y)) = \theta(k - (a-y) - (j-y)). \tag{A44}$$

Since the allele of at least one parent genotype needs to coincide with the allele of the offspring genotype, the number of 1-alleles that one can mutate is reduced by x. The same logic applies to the number of 0-alleles one can mutate, which is reduced by $i - x$. Finally in order to compute the recombination weight we have to sum over all possible combinations of distances (i, j) which are restricted due to Eq (A39) to be in the range $0 \leq i + j \leq L$. For efficient computation one should avoid double counting of ordered pairs (i, j) and (j, i) which yield the same contribution to the recombination weight. Combining these considerations leads to a more efficient expression for the recombination weight on the mesa landscape,

$$\begin{aligned}\lambda(L,k,a,r) =& \frac{1}{2^L} \sum_{i=0}^{\lfloor L/2 \rfloor} \sum_{j=i}^{L-i} \sum_{x=\max(0,i+a-L)}^{\min(i,a)} \binom{a}{x} \binom{L-x}{i-x} \theta(k+2x-a-i) \times \\ & \sum_{y=\max(0,j+a-L+i-x)}^{\min(j,a-x)} \binom{a-x}{y} \binom{L+x-a-i}{j-y} \theta(k+2y-a-j) \left[\frac{r}{2^{i+j}} (2-\delta_{ij}) + (1-r)\delta_{i0} \right],\end{aligned} \tag{A45}$$

where $\lfloor z \rfloor$ stands for the greatest integer that is less than or equal to z. As explained in the main text $\lambda(L, k, a, r)$ depends linearly on the recombination rate r. We use Eq (A45) for numerical calculations.

VI. MAXIMAL ROBUSTNESS IN THE PERCOLATION LANDSCAPE

To estimate the number of viable neighbors of a genotype in the percolation landscape in the limit of large L, we start from the observation that the expected number of genotypes with k viable neighbors is

$$\mathbb{E}(n_k) = 2^L \binom{L}{k} p^k (1-p)^{L-k} \sim \exp[L(\ln 2 - s_p(k/L))], \tag{A46}$$

where

$$s_p(z) = -z \ln(p) - (1-z)\ln(1-p) + z\ln(z) + (1-z)\ln(1-z) \tag{A47}$$

is the large deviation function of the binomial distribution [8]. For a given p, there is thus a value $z^*(p)$ defined by $s_p(z^*) = \ln 2$ such that, for $L \to \infty$, $\mathbb{E}(n_k) \to \infty$ if $k < z^* L$ and $\mathbb{E}(n_k) \to 0$ if $k > z^* L$. Using standard probabilistic

arguments this can be shown to imply that genotypes with k neighbors are present (absent) with probability 1 if $k < z^*L$ ($k > z^*L$), respectively. Thus the expected maximal robustness is $\overline{m}_{\max} = z^*$. Since $s_p(1) = \ln(1/p)$, $z^* = 1$ for $p \geq \frac{1}{2}$. Fig A2 compares the asymptotic behavior of $\overline{m}_{\max}$ for $L \to \infty$ to simulation results at finite L.

[1] URL `http://networkx.readthedocs.io/en/networkx-1.11/`.
[2] M. A. Nowak, M. C. Boerlijst, J. Cooke, and J. Maynard Smith, Nature **388**, 167 (1997).
[3] R. A. Neher, B. I. Shraiman, and D. S. Fisher, Genetics **184**, 467 (2010).
[4] G. Szegö, *Orthogonal polynomials* (American Mathematical Society, Providence, Rhode Island, 1975), 4th ed.
[5] A. Wolff and J. Krug, Phys. Biol. **6**, 036007 (2009).
[6] J. Hermisson, O. Redner, H. Wagner, and E. Baake, Theor. Pop. Biol. **62**, 9 (2002).
[7] M. C. Boerlijst, S. Bonhoeffer, and M. A. Nowak, Proc. Biol. Sci. **263**, 1577 (1996).
[8] D. Sornette, *Critical Phenomena in Natural Sciences* (Springer, Berlin, 2000).
