# Supplementary material for: Recombination and mutational robustness in neutral fitness landscapes
Source: PLoS Comput Biol. 2019 Aug 15;15(8):e1006884. doi: 10.1371/journal.pcbi.1006884 (PMC6711544; doi:10.1371/journal.pcbi.1006884)
Supplement: S1 Fig — The figure shows the entropy of the genotype frequency distribution in the two-locus model defined as S=−∑σfσ*ln(fσ*). For small mutation rates the strongly recombining population primarily consists of a single genotype, which implies that S → 0. (PDF) [file pcbi.1006884.s002.pdf]

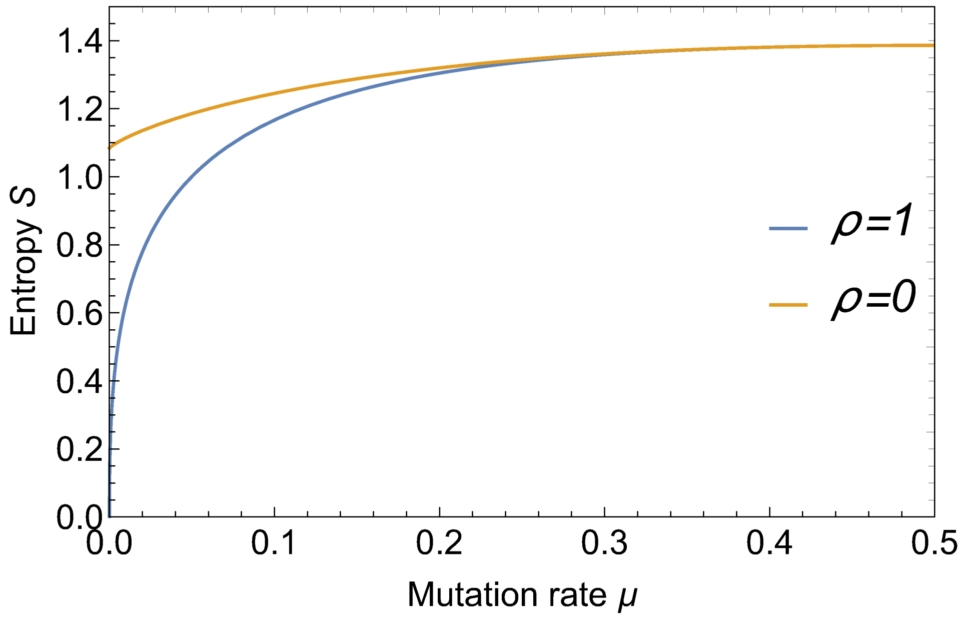

FIG. S1. **Population heterogeneity decreases with increasing recombination rate.** The figure shows the entropy of the genotype frequency distribution in the two-locus model defined as $S = -\sum_\sigma f^*_\sigma \ln(f^*_\sigma)$. For small mutation rates the strongly recombining population primarily consists of a single genotype, which implies that $S \to 0$.
