# Supplementary material for: Recombination and mutational robustness in neutral fitness landscapes
Source: PLoS Comput Biol. 2019 Aug 15;15(8):e1006884. doi: 10.1371/journal.pcbi.1006884 (PMC6711544; doi:10.1371/journal.pcbi.1006884)
Supplement: S2 Fig — The figure compares the analytic approximations in Eqs (29) and (30) to the numerical solution of the stationary genotype frequency distribution for the communal recombination scheme. The two panels show the mutational robustness as a function of the genome-wide mutation rate in linear (A) and double-logarithmic (B) scales, respectively. The parameters of the mesa landscape are L = 30 and k = 3. (PDF) [file pcbi.1006884.s003.pdf]

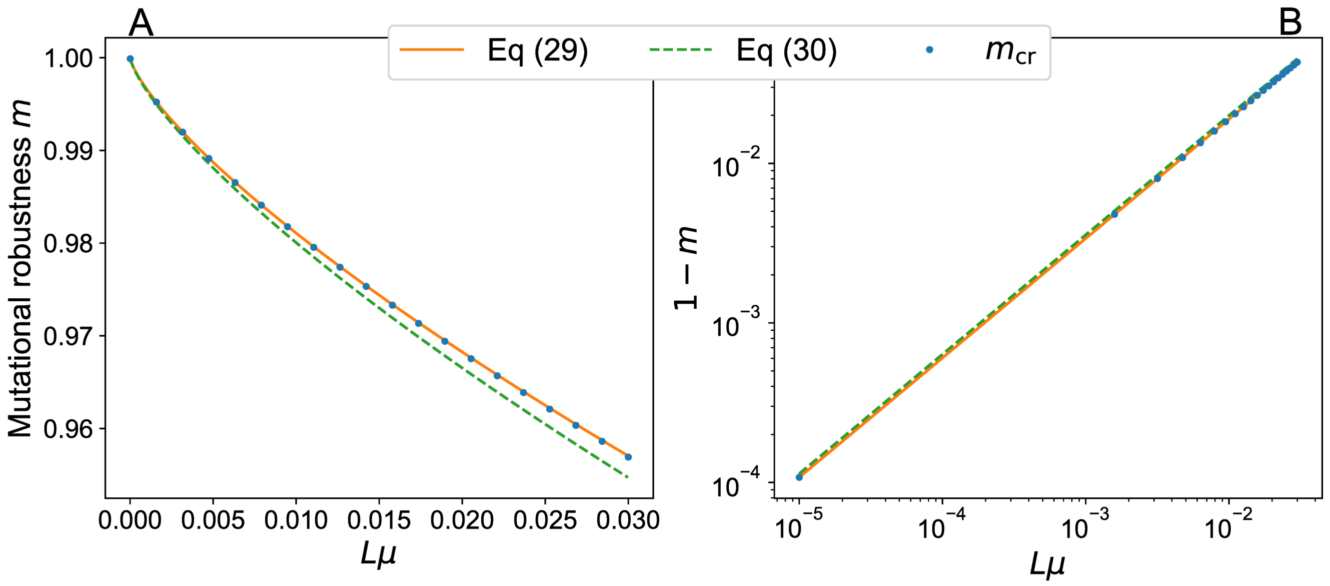

FIG. S2. **Mutational robustness for the mesa landscape with communal recombination.** The figure compares the analytic approximations in Eqs (29) and (30) to the numerical solution of the stationary genotype frequency distribution for the communal recombination scheme. The two panels show the mutational robustness as a function of the genome-wide mutation rate in linear (A) and double-logarithmic (B) scales, respectively. The parameters of the mesa landscape are $L = 30$ and $k = 3$.
