# Supplementary material for: Recombination and mutational robustness in neutral fitness landscapes
Source: PLoS Comput Biol. 2019 Aug 15;15(8):e1006884. doi: 10.1371/journal.pcbi.1006884 (PMC6711544; doi:10.1371/journal.pcbi.1006884)
Supplement: S3 Fig — The figure compares the analytic results for communal recombination (mcr) with numerical data obtained using uniform crossover (muc) and one-point crossover (mopc) at r = 1. The landscape parameters are L = 5, k = 2 and robustness is plotted as a function of the genome-wide mutation rate Lμ. (A) Mutational robustness on linear scales. (B) Double-logarithmic plot of 1 − m vs. Lμ, illustrating the power-law behavior 1 − m ∼ (Lμ)b with the exponent b = k/(k + 1) = 2/3 predicted by the analysis of the communal recombination model. (PDF) [file pcbi.1006884.s004.pdf]

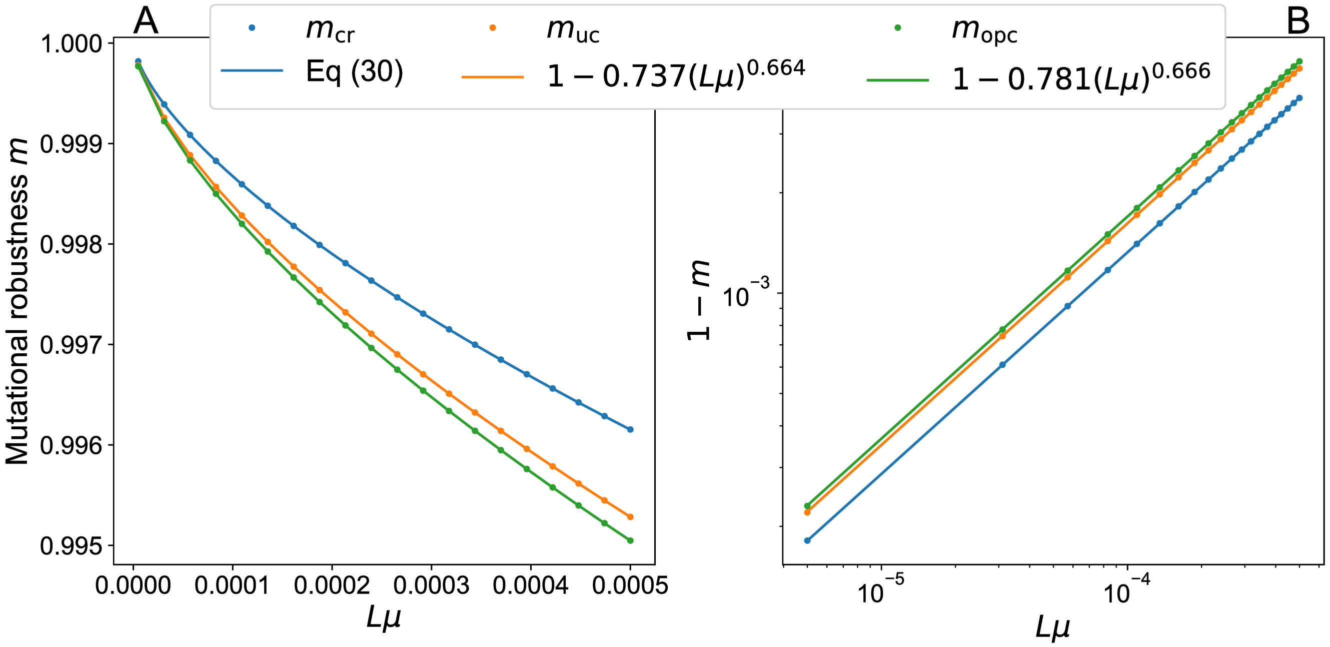

FIG. S3. **Mutational robustness in a mesa landscape with different recombination schemes.** The figure compares the analytic results for communal recombination (m_{cr}) with numerical data obtained using uniform crossover (m_{uc}) and one-point crossover (m_{opc}) at $r = 1$. The landscape parameters are $L = 5$, $k = 2$ and robustness is plotted as a function of the genome-wide mutation rate $L\mu$. (A) Mutational robustness on linear scales. (B) Double-logarithmic plot of $1 - m$ vs. $L\mu$, illustrating the power-law behavior $1 - m \sim (L\mu)^b$ with the exponent $b = k/(k + 1) = 2/3$ predicted by the analysis of the communal recombination model.
