# Supplementary material for: Recombination and mutational robustness in neutral fitness landscapes
Source: PLoS Comput Biol. 2019 Aug 15;15(8):e1006884. doi: 10.1371/journal.pcbi.1006884 (PMC6711544; doi:10.1371/journal.pcbi.1006884)
Supplement: S4 Fig — The figure compares the analytic predictions in Eqs (35) and (36) to the numerical solution for the genotype frequency distribution in the absence of recombination. The two panels show the mutational robustness (A) after selection and (B) after mutation as a function of the scaled mesa width x0 = k/L for L = 1000 and U = 0.01. (PDF) [file pcbi.1006884.s005.pdf]

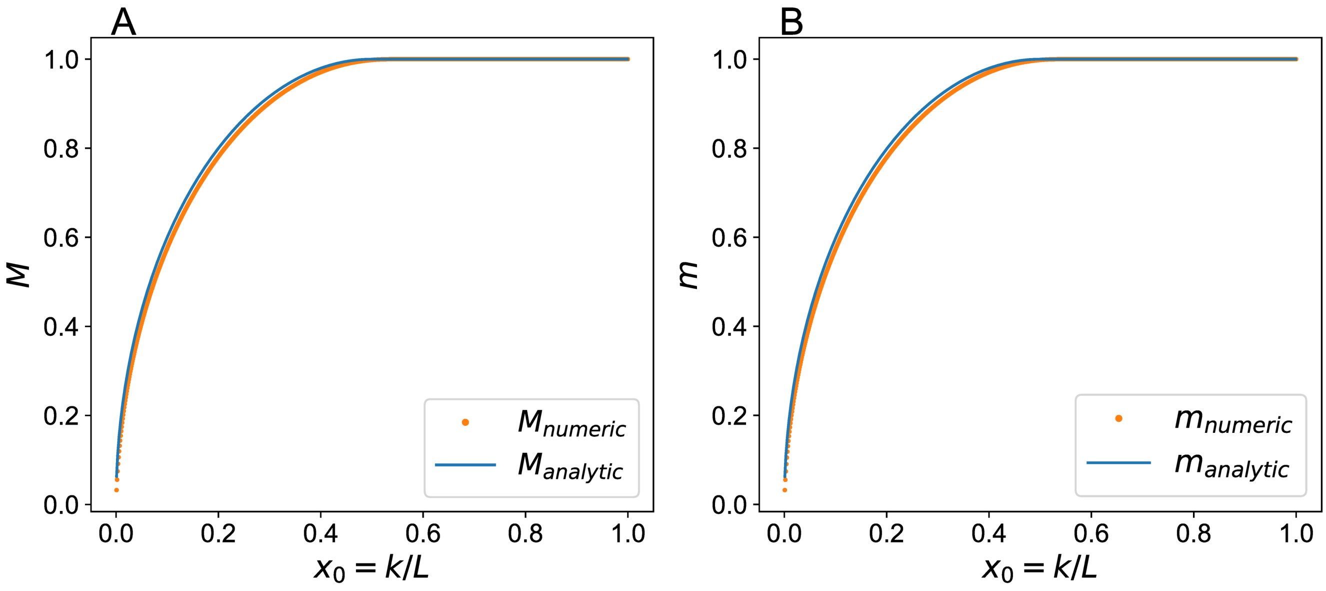

FIG. S4. **Mutational robustness for the mesa landscape in the absence of recombination.** The figure compares the analytic predictions in Eqs (35) and (36) to the numerical solution for the genotype frequency distribution in the absence of recombination. The two panels show the mutational robustness (A) after selection and (B) after mutation as a function of the scaled mesa width $x_0 = k/L$ for $L = 1000$ and $U = 0.01$.
