# Supplementary material for: Recombination and mutational robustness in neutral fitness landscapes
Source: PLoS Comput Biol. 2019 Aug 15;15(8):e1006884. doi: 10.1371/journal.pcbi.1006884 (PMC6711544; doi:10.1371/journal.pcbi.1006884)
Supplement: S5 Fig — Numerical results for communal recombination (mcr) and no recombination (mnr) are shown as dots. The mutational robustness m0 of a uniformly distributed population, given by Eq (37), as well as the analytic expressions Eqs (30) and (36) are depicted as lines. (A) Robustness as a function of mutation rate U = Lμ for a landscape with L = 1000 and k = 10. (B) Robustness as a function of mesa width k at fixed L = 1000 and U = Lμ = 0.01. (C) Robustness as a function of genome length L at fixed k = 10 and U = 0.01. (D) Robustness as a function of genome length L at fixed k = 10 and μ = 0.001. (PDF) [file pcbi.1006884.s006.pdf]

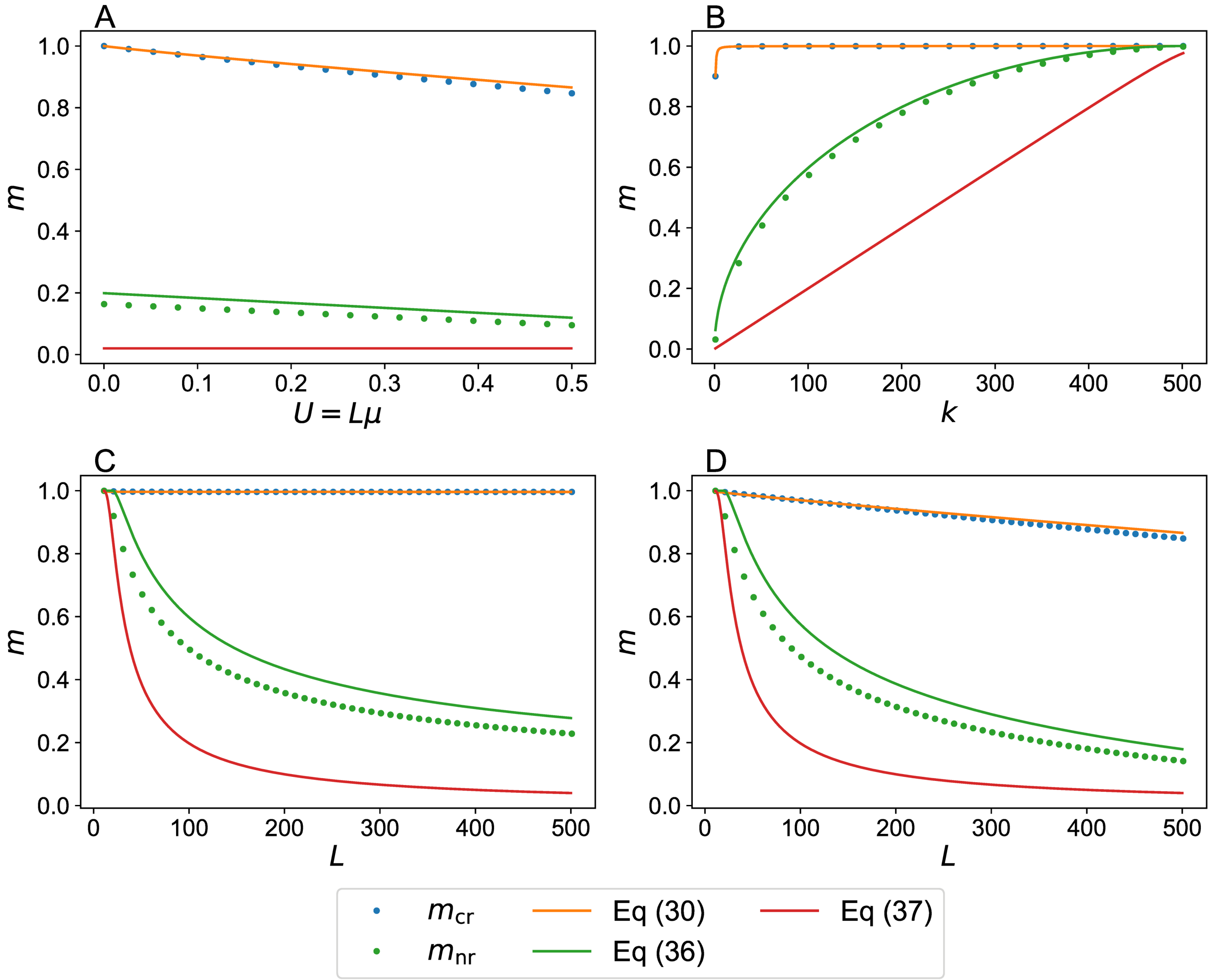

FIG. S5. **Mutational robustness in mesa landscapes with and without recombination**. Numerical results for communal recombination ($m_{\rm cr}$) and no recombination ($m_{\rm nr}$) are shown as dots. The mutational robustness m_0 of a uniformly distributed population, given by Eq (37), as well as the analytic expressions Eqs (30) and (36) are depicted as lines. (A) Robustness as a function of mutation rate $U = L\mu$ for a landscape with $L = 1000$ and $k = 10$. (B) Robustness as a function of mesa width k at fixed $L = 1000$ and $U = L\mu = 0.01$. (C) Robustness as a function of genome length L at fixed $k = 10$ and $U = 0.01$. (D) Robustness as a function of genome length L at fixed $k = 10$ and $\mu = 0.001$.
