# Supplementary material for: Recombination and mutational robustness in neutral fitness landscapes
Source: PLoS Comput Biol. 2019 Aug 15;15(8):e1006884. doi: 10.1371/journal.pcbi.1006884 (PMC6711544; doi:10.1371/journal.pcbi.1006884)
Supplement: S6 Fig — The parameters of the mesa landscape are L = 100 and k = 10. For r = 0 the recombination weight is directly proportional to the fitness and hence equal for all viable genotypes. Already small rates of recombination are sufficient to redistribute the recombination weight such that the weight of genotypes with small Hamming distance is strongly enhanced. Beyond d = 20 the recombination weight is identically zero, since the recombinant of two viable genotypes cannot carry more than 2k mutations. (PDF) [file pcbi.1006884.s007.pdf]

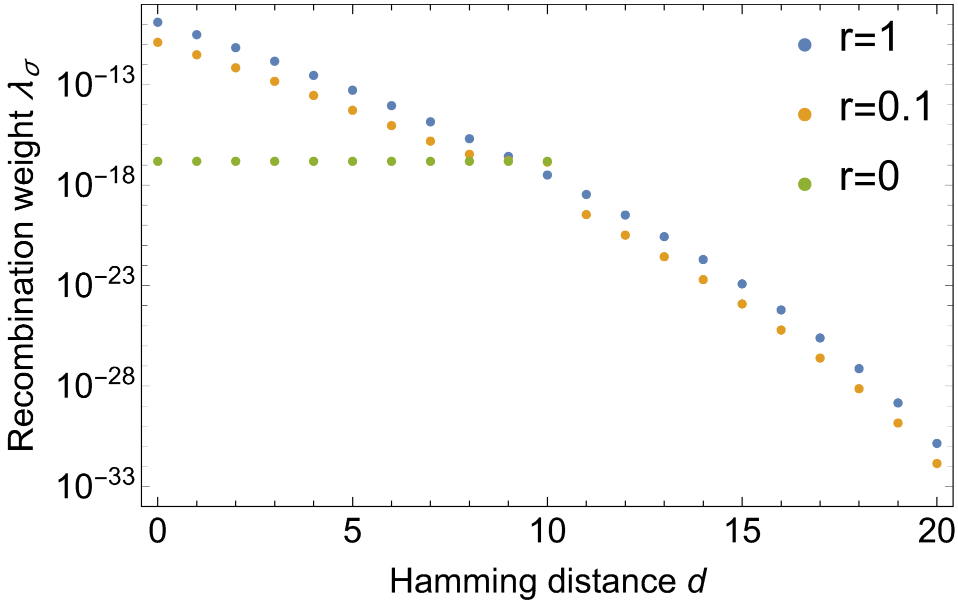

FIG. S6. **Recombination weight in a mesa landscape.** The parameters of the mesa landscape are $L = 100$ and $k = 10$. For $r = 0$ the recombination weight is directly proportional to the fitness and hence equal for all viable genotypes. Already small rates of recombination are sufficient to redistribute the recombination weight such that the weight of genotypes with small Hamming distance is strongly enhanced. Beyond $d = 20$ the recombination weight is identically zero, since the recombinant of two viable genotypes cannot carry more than $2k$ mutations.
