# Supplementary material for: Recombination and mutational robustness in neutral fitness landscapes
Source: PLoS Comput Biol. 2019 Aug 15;15(8):e1006884. doi: 10.1371/journal.pcbi.1006884 (PMC6711544; doi:10.1371/journal.pcbi.1006884)
Supplement: S7 Fig — The figure compares the mutational robustness of non-recombining (r = 0) and recombining (r = 1) populations on individual realizations of the percolation model with L = 6 and three values of p. In order to obtain different stationary states we used localized initial population distributions of the form fτ(0) = δτσ for all genotypes with mutational robustness mσ ≠ 0 and propagated them until stationarity. Since the stationary populations are usually highly concentrated for large r and small μ, this is a natural choice in order to access all stationary states. Each data point represents the robustness of the recombining population m(r = 1) for a particular stationary state. Data points within the same landscape are plotted above the corresponding unique robustness of the non-recombining population m(r = 0) and connected by a vertical line. The orange crosses show the average over all initial conditions. (PDF) [file pcbi.1006884.s008.pdf]

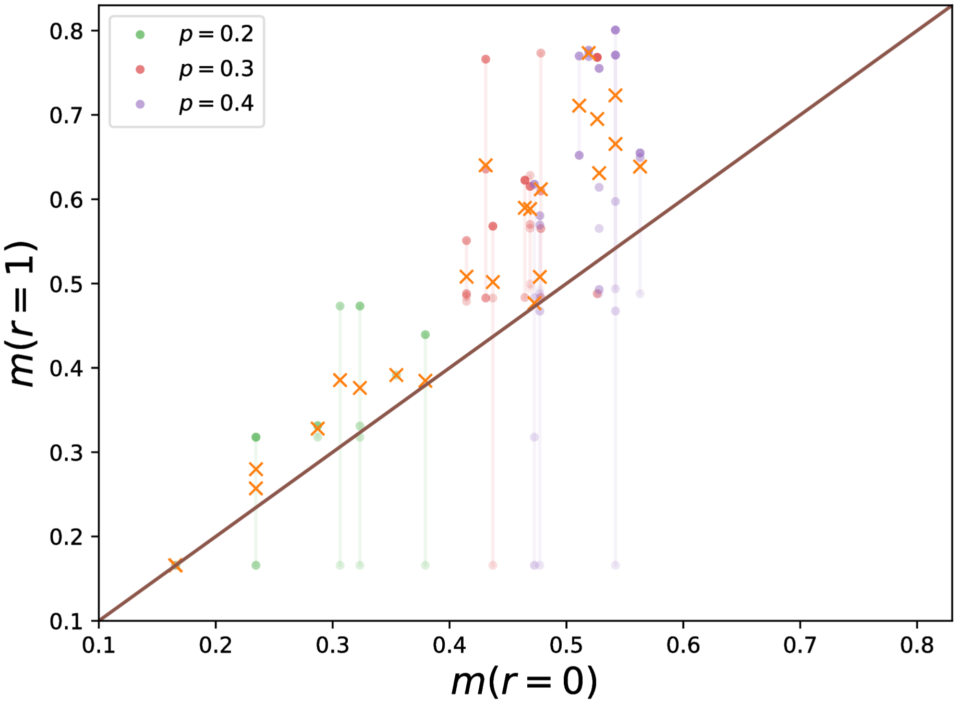

FIG. S7. **Mutational robustness for different stationary states within a percolation landscape**. The figure compares the mutational robustness of non-recombining ($r = 0$) and recombining ($r = 1$) populations on individual realizations of the percolation model with $L = 6$ and three values of p. In order to obtain different stationary states we used localized initial population distributions of the form $f_\tau(0) = \delta_{\tau\sigma}$ for all genotypes with mutational robustness $m_\sigma \neq 0$ and propagated them until stationarity. Since the stationary populations are usually highly concentrated for large r and small μ, this is a natural choice in order to access all stationary states. Each data point represents the robustness of the recombining population $m(r = 1)$ for a particular stationary state. Data points within the same landscape are plotted above the corresponding unique robustness of the non-recombining population $m(r = 0)$ and connected by a vertical line. The orange crosses show the average over all initial conditions.
