# Supplementary material for: Recombination and mutational robustness in neutral fitness landscapes
Source: PLoS Comput Biol. 2019 Aug 15;15(8):e1006884. doi: 10.1371/journal.pcbi.1006884 (PMC6711544; doi:10.1371/journal.pcbi.1006884)
Supplement: S8 Fig — Mutational robustness is computed for 200 randomly generated sea-cliff landscapes with parameters L = 6, d< = 1 and d> = 5, and the results are averaged to obtain m¯(r). The mutation rate is μ = 0.001. (PDF) [file pcbi.1006884.s009.pdf]

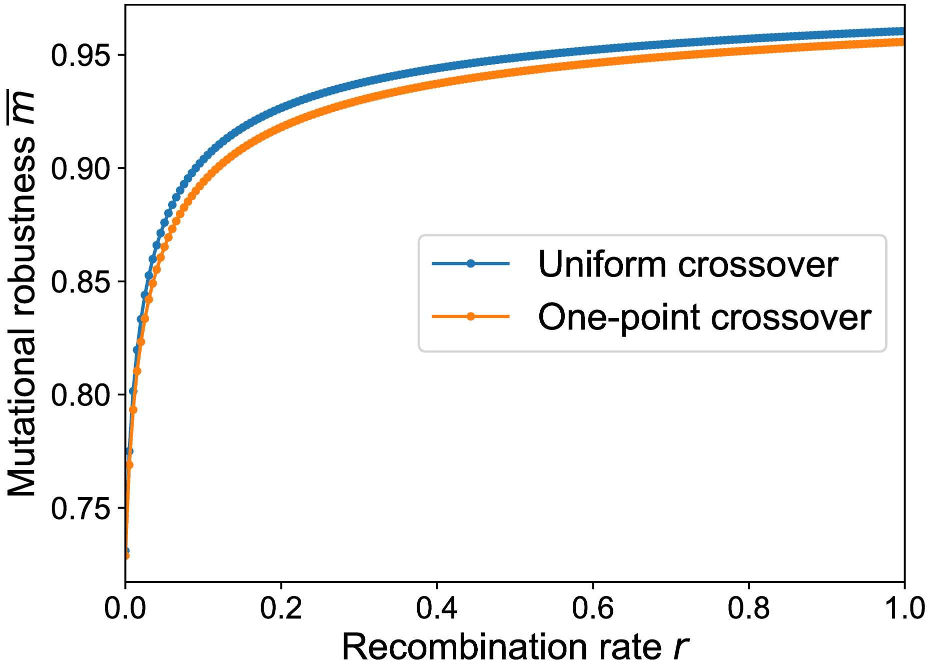

FIG. S8. **Average mutational robustness in the sea-cliff landscape as a function of recombination rate.** Mutational robustness is computed for 200 randomly generated sea-cliff landscapes with parameters $L = 6$, $d_< = 1$ and $d_> = 5$, and the results are averaged to obtain $\overline{m}(r)$. The mutation rate is $\mu = 0.001$.
