# Supplementary material for: Recombination and mutational robustness in neutral fitness landscapes
Source: PLoS Comput Biol. 2019 Aug 15;15(8):e1006884. doi: 10.1371/journal.pcbi.1006884 (PMC6711544; doi:10.1371/journal.pcbi.1006884)
Supplement: S9 Fig — The mutational robustness and the population-averaged fitness in the stationary state are computed as a function of recombination rate by evolving the population from a uniform initial genotype distribution at mutation rate μ = 0.005. Jumps mark changes in the most populated genotype. (PDF) [file pcbi.1006884.s010.pdf]

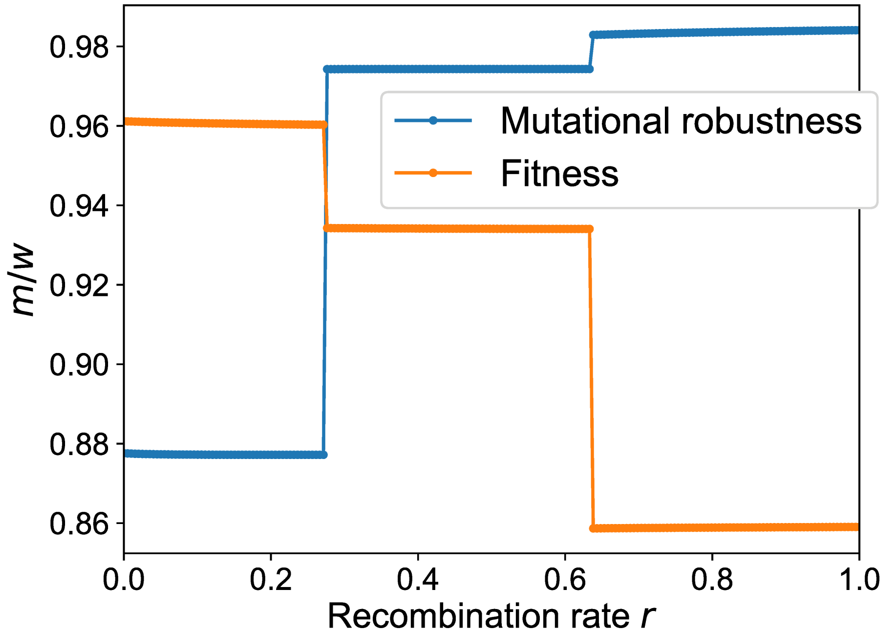

FIG. S9. **Mutational robustness and average fitness in the empirical *A. niger* fitness landscape.**. The mutational robustness and the population-averaged fitness in the stationary state were computed as a function of recombination rate by evolving the population from a uniform initial genotype distribution at mutation rate $\mu = 0.005$. Jumps mark changes in the most populated genotype.
