# Supplementary material for: Recombination and mutational robustness in neutral fitness landscapes
Source: PLoS Comput Biol. 2019 Aug 15;15(8):e1006884. doi: 10.1371/journal.pcbi.1006884 (PMC6711544; doi:10.1371/journal.pcbi.1006884)
Supplement: S10 Fig — This landscape is similar to the mesa landscape but includes an inner critical radius within which genotypes are lethal. In this example the inner radius is chosen to be 1 such that only the wild type is lethal. The outer radius is 2 and the sequence length is L = 7. The recombination rate is r = 1 and the mutation rate is μ = 0.001. The frequencies fn of the stationary state at the same Hamming distance n are lumped together. The population is concentrated at distance 1 which is most robust since only one point mutation is lethal, but the recombination center coincides with the lethal wild type. This example shows that the correlation between recombination weight and mutational robustness depends on the topology of the neutral network. (PDF) [file pcbi.1006884.s011.pdf]

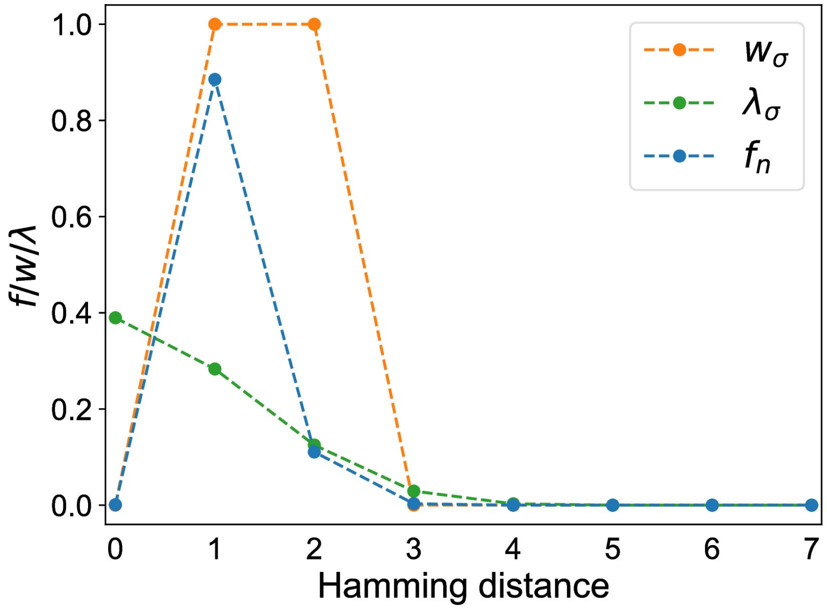

FIG. S10. **Recombination on an atoll landscape**. This landscape is similar to the mesa landscape but includes an inner critical radius within which genotypes are lethal. In this example the inner radius is chosen to be 1 such that only the wild type is lethal. The outer radius is 2 and the sequence length is $L = 7$. The recombination rate is $r = 1$ and the mutation rate is $\mu = 0.001$. The frequencies f_n of the stationary state at the same Hamming distance n are lumped together. The population is concentrated at distance 1 which is most robust since only one point mutation is lethal, but the recombination center coincides with the lethal wild type. This example shows that the correlation between recombination weight and mutational robustness depends on the topology of the neutral network.
