# Supplementary material for: Recombination and mutational robustness in neutral fitness landscapes
Source: PLoS Comput Biol. 2019 Aug 15;15(8):e1006884. doi: 10.1371/journal.pcbi.1006884 (PMC6711544; doi:10.1371/journal.pcbi.1006884)
Supplement: S11 Fig — The figure shows the mutational robustness in a mesa landscape with parameter L = 6, k = 2 as a function of mutation rate. The finite population results were obtained using Wright-Fisher dynamics for N = 1000 individuals. For small mutation rates such that NμL ≪ 1 the monomorphic population performs a random walk among viable genotypes, which leads to the uniform mutational robustness m0 given by Eq (37) (green dashed line). In this regime recombination cannot have any effect. For NμL > 1 the robustness rises sharply to the value predicted by the infinite population approach. At the maximal mutation rate μ = 0.5 the population is uniformly distributed among all (lethal or viable) genotypes after the mutation step and recombination has again no effect. (PDF) [file pcbi.1006884.s012.pdf]

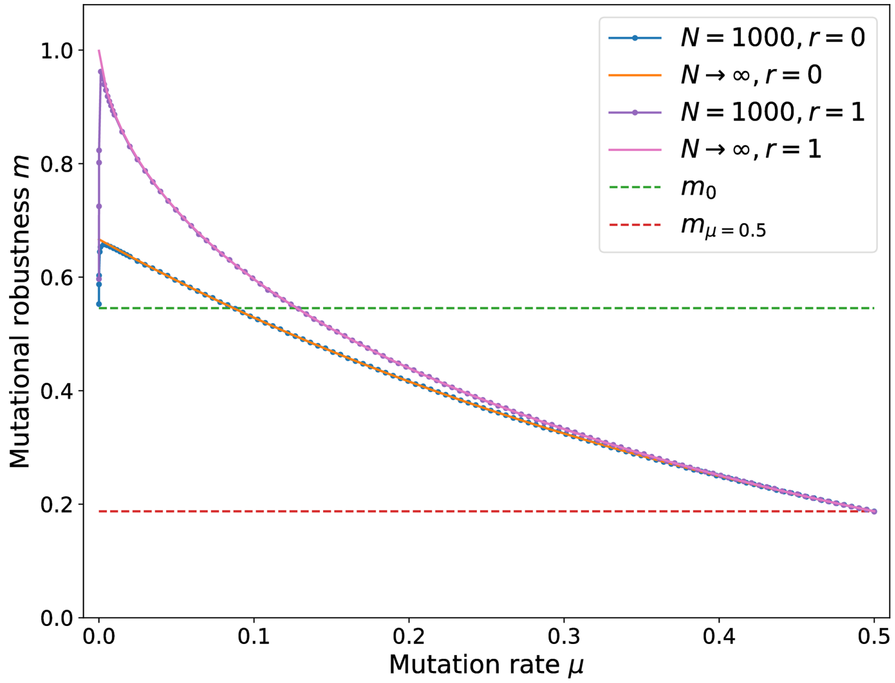

FIG. S11. **Finite population size effects**. The figure shows the mutational robustness in a mesa landscape with parameter $L = 6, k = 2$ as a function of mutation rate. The finite population results were obtained using Wright-Fisher dynamics for $N = 1000$ individuals. For small mutation rates such that $N\mu L \ll 1$ the monomorphic population performs a random walk among viable genotypes, which leads to the uniform mutational robustness m_0 given by Eq (37) (green dashed line). In this regime recombination cannot have any effect. For $N\mu L > 1$ the robustness rises sharply to the value predicted by the infinite population approach. At the maximal mutation rate $\mu = 0.5$ the population is uniformly distributed among all (lethal or viable) genotypes after the mutation step and recombination has again no effect.
